# Monitoring of canonical BMP and Wnt activities during postnatal stages of mouse first molar root formation

**DOI:** 10.1590/1678-7757-2021-0281

**Published:** 2021-12-13

**Authors:** Jia WANG, Shujun RAN, Bin LIU, Shensheng GU

**Affiliations:** 1 Shanghai Jiao Tong University School of Medicine Ninth People’s Hospital Shanghai China Shanghai Jiao Tong University, School of Medicine, Ninth People’s Hospital, Department of Endodontics and Operative Dentistry, Shanghai, China.; 2 Tulane University Department of Cell and Molecular Biology New Orleans LA USA Tulane University, Department of Cell and Molecular Biology, New Orleans, LA, USA.

**Keywords:** BMP signaling activity, Molar, Odontogenesis, Tooth development, Wnt signaling activity

## Abstract

**Objective:**

This study aimed to explore the precise temporospatial distributions of bone morphogenetic protein (BMP) and Wnt signaling pathways during postnatal development of mammalian tooth roots after the termination of crown morphogenesis.

**Methodology:**

A total of two transgenic mouse lines, BRE-LacZ mice and BAT-gal mice, were undertaken. The mice were sacrificed on every postnatal (PN) day from PN 3d up to PN 21d. Then, the first lower molars were extracted, and the dissected mandibles were stained with 5-bromo-4-chloro-3-indolyl-β-d-galactopyranoside (X-gal) and fixed. Serial sections at 10 µm were prepared after decalcification, dehydration, and embedding in paraffin.

**Results:**

We observed BMP/Smads and Wnt/β-catenin signaling activities in the dental sac, dental pulp, and apical papilla with a certain degree of variation. The position of activation of the BMP/Smad signaling pathway was located more coronally in the early stage, which then gradually expanded as root elongation proceeded and was associated with blood vessels in the pulp and developing complex apical tissues in the later stage. However, Wnt/β-catenin signaling was highly concentrated in the mesenchyme below the cusps in the early stage, gradually expanded to regions around the root in the transition/root stage, and then disappeared entirely in the later stage.

**Conclusions:**

These results further confirmed the participation of both BMP and Wnt canonical signaling pathways in tooth root development, as well as formed the basis for future studies on how precisely integrated signaling pathways regulate root morphogenesis and regeneration.

## Introduction

Analogous to the ectodermal organogenesis, a series of reciprocal and sequential epithelial-mesenchymal interactions are essential for tooth root development, which occurs after crown formation.^1^ Multiple signaling cascades have implicated in these processes to guide root development in different stages.^2^ The outer enamel epithelium is confluent with the inner enamel epithelium at the cervical loop, as well as grows in the apical direction forming the bilayer of an epithelial extension called Hertwig’s epithelial root sheath (HERS), a transient structure that occurs before the complete formation of the tooth root.^3^ The formed HERS extends apically and envelops the dental papilla to form a barrier separating from the dental sac. The HERS is known to induce the differentiation of odontoblasts and cementoblasts, to guide root growth, and to determine the number of tooth roots. HERS disintegration orchestrates the deposition of root dentin, which subsequently initiates dental sac cell cementogenesis onto the dentin surface.^4,5^ Moreover, it may also participate in cementum formation via epithelial-mesenchymal transition.^6,7^ Thus, HERS plays a vital role in root development.

BMP and Wnt signaling pathways are crucial for root formation, including cell fate, growth, and patterning. The BMP–Smad4–Shh–Gli1–Sox2 signaling cascade worked in root development, where the disruption of BMP signaling in the dental epithelium causes delays in HERS formation and affects the stem cell niche environment.^8^ Moreover, the ablation of *Smad4,* the key intracellular mediator of the TGF-β/BMP signaling pathway, in odontoblasts disrupts odontoblast differentiation and causes abnormal root odontogenesis.^9,10^ Previous studies also revealed the essential role of Wnt signaling during root formation; we detected the canonical Wnt activity in the mesenchyme and odontoblasts in *BAT-gal/ TOP-gal* reporter mice.^11,12^ Furthermore, recent reports showed that the disruption of β-catenin in immature odontoblasts generates molars without roots, whereas the constitutive stabilization of β-catenin can also lead to aberrant short roots with excessive cementum.^13,14^

The cervical loop structure of mouse molars disappears after crown formation, and then HERS is formed to guide root development postnatally, which is similar to human tooth organogenesis. Thus, the first lower mouse molar (M1) is an ideal model for studying tooth root development. In the past decades, the molecular regulatory network of early tooth morphogenesis was studied extensively, with research mainly concentrating on prenatal stages.^15,16^ However, the journey toward the functional tooth starts postnatally. Furthermore, a comprehensive study on BMP and Wnt signaling activity during postnatal mouse M1 morphogenesis remains elusive.

This study aimed to investigate postnatal M1 morphogenesis before eruption associated with active BMP and Wnt signaling distribution, matching with root development and odontogenesis. The *BRE-LacZ* reporter contains a BMP-response element (BRE) labeled by β-galactosidase, in which phosphorylated BMP Smad DNA-binding sites are originated with the mouse *Id1* promoter.^17^ Similarly, *BAT-gal* mouse is a Wnt-reporter transgenic strain that expresses β-galactosidase when the intracellular β-catenin is activated (mimicking the canonical Wnt signaling pattern).^18^ These *BRE-LacZ* or *BAT-gal* reporter mouse lines can identify sites where the BMP-Smads or Wnt/β-catenin signaling pathway is activated. They represent an opportunity to monitor BMP/Smad-dependent or Wnt/β-catenin-dependent signaling during M1 development. Therefore, we used *BRE-LacZ* and *BAT-gal* mice to monitor their activities in postnatal stages of tooth development to explore the potential roles of BMP and canonical Wnt/β-catenin signaling in late tooth morphogenesis. Indeed, the findings indicated that BMP and Wnt signaling pathways orchestrated an active network that regulated postnatal tooth development.

## Methodology

### Animals

Animal care and all experiments were conducted under the recommendations in the Guide for Care and Use of Laboratory Animals of the National Institutes of Health, approved by the Institutional Animal Care and Use Committee of Tulane University (Protocol #: 0184R5)

The day of birth was designated as “postnatal day (PN) 0.” Before the sacrifice, the tail biopsies from each mouse were determined by PCR-based genotyping. The generation and genotyping protocols of transgenic mice used in our study were described previously: *BRE-LacZ* mice^17^ and *BAT-gal* mice^18^. All studies involved CD-1 background mice. The mice were kept in a 12-hour light/dark period with a temperature of 22 ± 2^0^C and humidity of 45% ± 15% of the rooms.

#### Tissue preparation

Pups from mice of both sexes were sacrificed on every PN day from day 3 (PN3) up to PN21 (n: 5 pups). Extracted first lower molars and dissected mandibles were stained with X-gal, fixed, decalcified in 10% ethylene diaminetetraacetic acid/phosphate-buffered saline (PBS) solution (pH 7.4), dehydrated through a graded alcohol series, and embedded in paraffin. Serial sections of 10 µm were prepared. Counterstaining for sectioned samples was done with eosin. For general histological observations, the specimens were examined under a light microscope (Leica, Germany).

#### X-gal staining

*LacZ* expression was determined with X-gal staining, as described earlier.^19^ Briefly, the mandibular samples were fixed with 4% paraformaldehyde (PFA) in 0.2 mM glutaraldehyde in PBS for 10 min, as well as were processed for X-gal staining in the buffer (containing 1 mg/mL of X-gal) for 20 h at 37°C in the dark. For general observations, the specimens were examined under a stereomicroscope (Leica, Germany).

## Results

**Figure 1:**
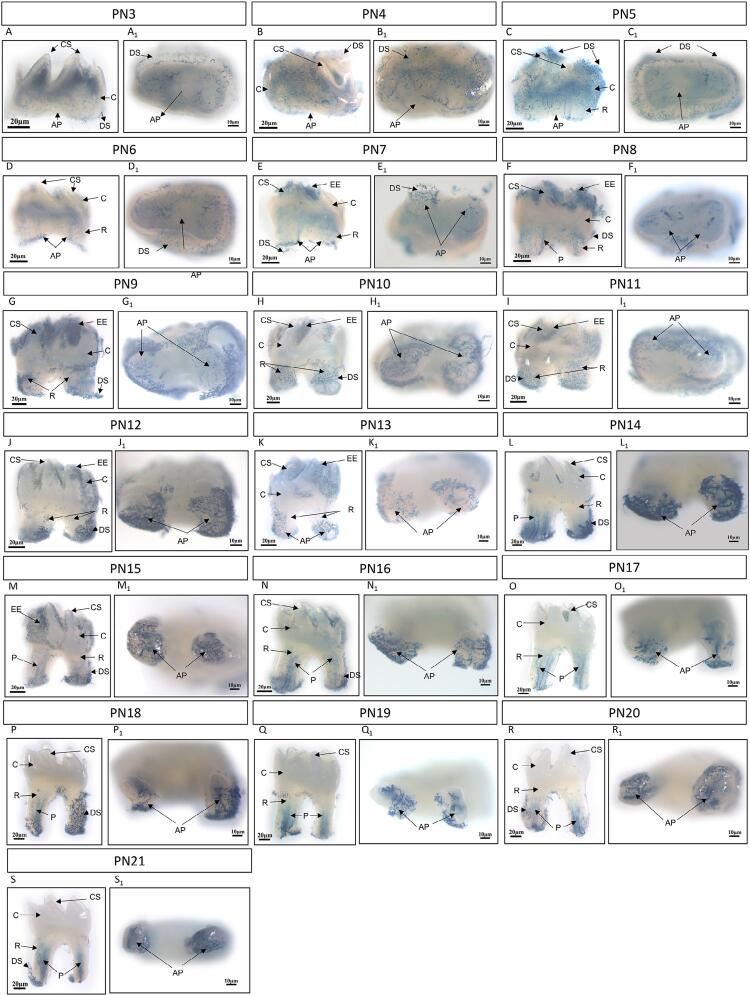
BRE-LacZ reporter activity during M1 postnatal development in mice ranging from postnatal day (PN) 3 to PN21. A whole-mount X-gal staining of M1 showed BRE-dependent BMP signaling in the buccal/lingual view (A, B, C, D, E, F, G, H, I, J, K, L, M, N, O, P, Q, R, and C) and in the apical view (A1, B1, C1, D1, E1, F1, G1, H1, I1, J1, K1, L1, M1, N1, O1, P1, Q1, R1, and S1). On PN3-PN5, active BMP/Smad signaling was identified in the coronal pulp and dental sac surrounding M1 (A–C) (A1–C1). On PN6–PN8, active BMP/Smad signaling could be observed in the dental papilla and root sheath (D–F) (D1–F1). On PN9–PN14, strong BMP/Smad signaling was observed in the root-forming regions, more specifically in the apical papilla and root sheath (G–L) (G1–L1). On PN15–PN21, the expression of BMP/Smad signaling was diminished around the root but existed in the apical region and along the ordered lining of blood vessels in the pulp (M–S) (M1–S1). (EE, enamel epithelium; DS, dental sac; CS, cusp; C, crown; R, root; AP, apical papilla; P, pulp).

**Figure 2:**
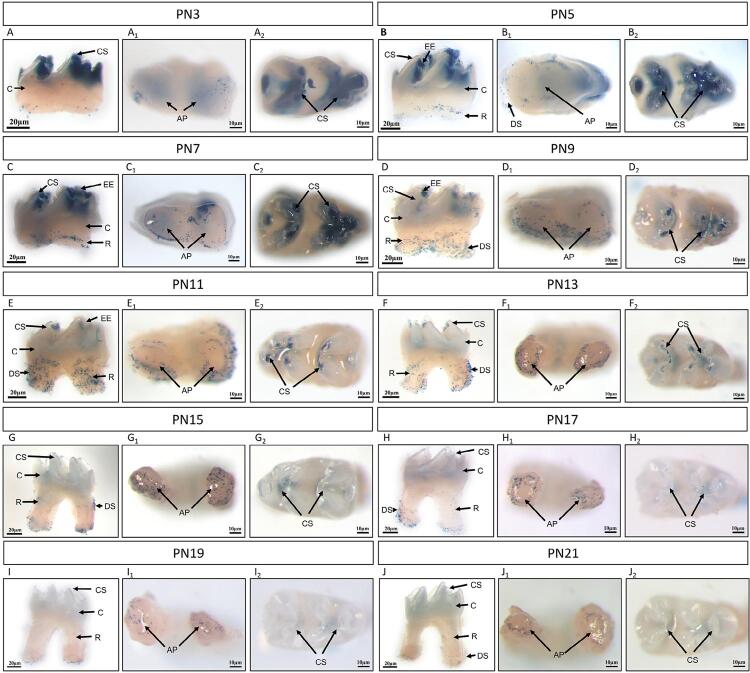
BAT-gal reporter activity during M1 postnatal development in mice ranging from PN3 to PN21. A whole-mount X-gal staining of M1 showed Wnt/β-catenin signaling in the buccal/ lingual view (A, B, C, D, E, F, G, H, I, and J), in the apical view (A1, B1, C1, D1, E1, F1, G1, H1, I1, and J1), and in the occlusal view (A2, B2, C2, D2, E2, F2, G2, H2, I2, and J2). On PN3 and PN5, active Wnt/β-catenin signaling was detected in the mesenchyme directly below the cusps (A–B) (A1–B1) (A2–B2). On PN7, active Wnt/β-catenin signaling was observed in the apical region, with a decrease in the area below the cusps (C–C2). During PN9–PN14, strong Wnt/β-catenin signaling was observed in the root-forming regions not in the crown, and more specifically in the apical papilla and root sheath (D–F) (D1–F1) (D2–F2). From PN15 to PN21, the expression of Wnt/β-catenin signaling was diminished mostly, but some expression was observed in the apical region (G–J) (G1–J1) (G2–J2). (EE, enamel epithelium; DS, dental sac; CS, cusp; C, crown; R, root; AP, apical papilla; P, pulp; CL, cervical loop)

The results showed that M1 crown development was completed on postnatal day 3 (PN3) (Figures 1A, 2A) and then root development began. By that time, primary odontogenesis transited to the zone of developing roots. HERS was formed from the cervical loop epithelium apically with inward growth. The development of the tooth root commenced on PN4 or PN5 (Figures 1B-1C, 2B), with the most apical side of HERS tending to curve inward and become narrow.

We used *BRE-LacZ* reporter mouse line to identify BMP activity in tooth development. β-galactosidase reporter gene expression identified the broad and dynamic BMP signaling activities during postnatal root development. For the early postnatal days, we harvested the M1 with the whole dental sac. Since the newly formed cusp was fragile and soft, it was difficult to separate the dental sac or epithelium from the crown until PN14 (Figures 1A–1C). The whole-mount X-gal staining results detected *BRE-LacZ* activity in the dental sac around the tooth, the pulp, and the apical papilla (around the apical foramen) in the initial stages. During the following stages of M1 morphogenesis (PN6–PN8), the teeth rapidly increased in size and length. *BRE-LacZ* activity became lower in the crown pulp but intensified in the root pulp, dental sac, and apical papilla (Figures 1D–1F). Between PN9 and PN14, significant root growth and further tooth mineralization occurred with crown calcification (Figures 1G–1L). Meanwhile, we established the eruption path for M1 and the tooth was ready to erupt into the oral cavity, to reach its occlusal position, and to perform its function after PN14 (data not shown). We detected strong X-gal staining at the dental sac around the root and the apical region (Figures 1G–1L). Furthermore, the enamel epithelium surrounding the crown showed positive staining when it transformed into the reduced enamel epithelium and subsequently fused with the oral epithelium (Figures 1G–1M). On PN15 and PN16, M1 developed almost completely; however, the apical foramen was still wide with dental papilla (Figures 1M–1N). Following, canonical BMP signaling activity decreased in the dental sac and crown pulp with the elongation of the root but intensified in the apical region (Figures 1L–1S). On PN21, M1 protruded into the oral cavity (data not shown), and root development was almost completed with long roots and narrow apical foramen (Figure 1S). The obvious activity of BMP/Smad-dependent signaling was only retained along the ordered lining of blood vessels in the pulp.

We traced Wnt/β-catenin signaling in the developing molar using the *BAT-gal* allele, a reporter mouse line that expressed β-galactosidase in the presence of activated β-catenin. On PN3, we established the enamel production. Different from BMP/Smad signaling activity, active Wnt/β-catenin signaling was restricted in the crown pulp region beneath the cusps and did not appear in the dental sac, root pulp, and apical papilla (Figures. 2A–A_2_). Wnt/β-catenin signaling activity was highly concentrated under the tip of the molar cusps during PN3–PN6, a stage before dentinogenesis in the root region (Figures 2A and 2B). We found some activated canonical Wnt signaling in the apical papilla and dental sac after rapid tooth growth and elongation on PN7 (Figures 2C–C_2_). Then, X-gal staining intensity reduced in the crown but increased in the apical area and dental sac (Figures 2C–2F). When M1 was ready to erupt on PN14, the intensity of X-gal staining was weak at the zones below the cusps (Figure 2F_2_). During later postnatal stages, the region with active Wnt/β-catenin signaling became gradually restricted to the crown and apical papilla, and the activity was barely detectable in the pulp (Figures 2G–2J).

**Figure 3:**
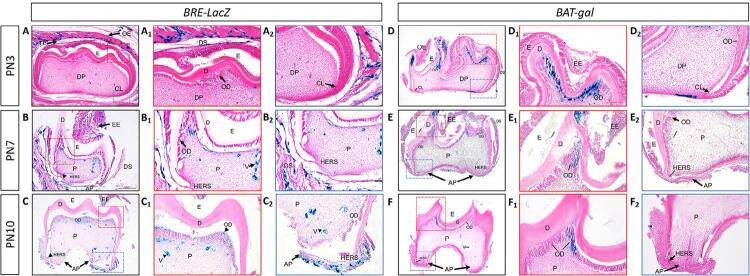
Selected histological sections of postnatal M1 with whole-mount X-gal staining at 10 µm thickness. Localization of active BMP/Smad signaling (A, B, and C, ×40) and Wnt/β-catenin signaling (D, E, and F, ×40) in M1 is shown. Panels A1, B1, C1, D1, E1, and F1 are high-magnification views (×100) of the red rectangular block as shown in panels A, B, C, D, E, and F, respectively. Panels A2, B2, C2, D2, E2, and F2 are high-magnification views (×100) of the blue rectangular block as shown in panels A, B, C, D, E, and F, respectively. On PN3, BMP/Smad-dependent signaling was primarily detected in the dental epithelium (A, A1, and A2); whereas active Wnt/β-catenin signaling was mainly expressed in the odontoblasts or pre-odontoblasts below the cusps and in the dental epithelium on the cusps; some expression was found in the apical region (D, D1, and D2). On PN7, BRE activity was detected in the pre-odontoblasts/odontoblasts in the crown pulp, blood vessels, and DAC (B, B1, and B2); Wnt/β-catenin activity was observed below the cusps, but none in the dental papilla and sac, nor in the vessels in the pulp (E, E1, and E2). On PN10, the same expression pattern was identified, associated with significant root elongation and advanced tooth mineralization (C, C1, and C2). Meanwhile, in BAT-gal mice, the number of X-gal-positive cells beneath the cusps decreased on PN10 (F, F1, and F2). (EE, enamel epithelium; OE, oral epithelium; DS, dental sac; CS, cusp; C, crown; R, root; DP, dental papilla; AP, apical papilla; P, pulp; CL, cervical loop; E, enamel; D, dentin; OD, odontoblast; V, vessel; HERS, Hertwig’s epithelial root sheath)

We chose several typical postnatal tooth development stages (PN3, PN7, and PN10) to assess the histomorphology. Figure 3A showed that we primarily detected BMP/Smad-dependent signaling in the dental epithelium on PN3. Then, BMP signaling activity moved downward to the cervical loop, confining to the HERS and apical papilla. On PN7, we observed BRE activity in the odontoblasts in the crown pulp and blood vessels (Figure 3B). Developing apical complex (DAC) comprises the apical papilla, dental sac, and HERS, regarded as inseparable integrity. On PN10, the level of BMP/Smad signaling activity was higher in the odontoblasts of the dental pulp, vascular tissues, and DAC, which was associated with significant root elongation and advanced tooth mineralization (Figure 3C). We observed Wnt/β-catenin signaling activity in a different manner. On PN3, we detected a high β–galactosidase expression level in the mesenchymal cells under the cusps where the odontoblast or pre-odontoblast existed (Figure 3D). We associated the expression with the tip of the molar cusps but excluded from the ameloblasts. With continuing root development, the number of X-gal-stained cells beneath the cusps decreased. On PN7 and PN10, we could observe some X-gal-stained odontoblasts below the cusps, but none in the dental papilla and sac, nor in the vessels in the pulp (Figures 3E and 3F). Despite the limitation to capture all and precise sites of active BMP or Wnt signaling, these results revealed the dynamic activities of BMP and Wnt signaling pathways in a spatiotemporal manner during postnatal tooth root morphogenesis.

## Discussion

Tooth morphogenesis is a well-orchestrated process governed by many signaling pathways between resident/migratory cells and the extracellular matrix. Multiple signaling pathways and numerous transcription factors have been illustrated in the molar root development; however, few concerns regarding their dynamic expression postnatally remain. Contrary to incisor development, mouse molar development is similar to that in humans, including coronal morphogenesis followed by the initiation of root development and later root elongation. In mice, tooth root development begins after birth and lasts for almost 21 days. Therefore, mouse M1 is an ideal animal model in mammalian odontogenesis research. Currently, the underlying mechanisms that control root formation during odontogenesis are yet to be determined. Many studies showed that BMP and Wnt ligands persist throughout tooth root formation. However, the dynamic pattern of active BMP and Wnt signaling is still not well understood. To allow screening for the strength of the two signals, we chose to derive two transgenic reporter strains, *BRE-LacZ* and *BAT-gal* mice. Both strains were associated with β-galactosidase. X-gal staining in *BRE-LacZ* reporter correlated with the nuclear accumulation of phosphorylated SMAD 1/5/8 (BMP activation), whereas *BAT-gal* transgenic strain in the presence of activated β-catenin (mimics the pattern of Wnt/β-catenin signaling) *e*xhibited β-gal activity. This study revealed the spatiotemporal activities of both BMP/Smad and Wnt/β-catenin signaling pathways in the developing molar root.

Epithelial-mesenchymal interactions eventually contribute to the formation of dentin-pulp complex, cementum, and periodontium. The expression of BMP signaling molecules in both dental epithelium and mesenchyme is necessary in root development progresses.^4,20^ We observed that Bmp2 signaling is indispensable for proper enamel development and mineralization *in vivo.*^21^ The blockage of BMP signaling in the dental mesenchyme on PN3.5 resulted in impaired root development, whereas inhibiting BMP signaling in the dental epithelium before root growth certified that postnatal epithelial BMP signaling was dispensable in the regulation of root development.^22^ Similar to many other developmental processes, epithelial−mesenchymal interactions were previously demonstrated to be essential for tooth root development.^4^ Here, we showed that activated BMP signaling (positive X-gal staining) was specifically present in dental follicles and HERS at the early postnatal stage; then, the HERS became perforated, contributing to cementum regeneration and root formation (Figures 1 and 3). When M1 root formation was almost completed, BMP/Smad activity appeared restricted in the apical part of the root and in the pulp.

During molar root formation, BMP signaling seemed to influence odontogenesis in the light of dynamic spatial and temporal sites of the canonical BMP signaling activity. In developing M1, differentiated odontoblasts are parallel to the basement membrane after birth and then ready to secrete dentin matrix. Next, the rapid tooth formation and root growth originate at P6.^23^Figure 3 showed that activated BMP signaling (positive X-gal staining) was localized more coronally, correlating the BMP signaling with dental mesenchyme cells committed into odontoblasts. Furthermore, previous researchers revealed that Smad4-dependent BMP signaling acts a fundamental part in crown development, and non-Smad signaling may be useful for root dentin formation and elongation.^24^ During molar root formation, previous studies found that the dynamic spatiotemporal regulation of BMP activity might define the stem cell niche involved in root growth.^22^ In this study, we observed that the last zone of BMP activity was found in the vascular tissues in the pulp and apical papilla (Figure 1), consistent with the BMP/smad-dependent signaling vessel walls, and localized to endothelial cells.^25^ The apical papilla harbored stem cells that probably generated primary odontoblasts that were responsible for root dentin production.^26^ The results suggested that active BMP signaling was essential for the apical growth of the molar root.

Meanwhile, Wnt/β-catenin signaling is also crucial for root odontogenesis, as evidenced by the conditional inactivation of β-catenin in immature odontoblasts, which induces molars without roots.^13^ Moreover, we observed the phenotype of ablation of Wnt ligands or overexpression of the inhibitor *Dkk-1* in odontoblasts in the mandibular molars, with aberrantly short roots and dentin defects.^27,28^ Thus, Wnt signaling in root formation needs to be tightly controlled.^4,15^ In this study, we also observed regions with active Wnt/β-catenin signaling in the enamel knot and the underlying mesenchyme in the initial postnatal stages. This could be attributed to the expression of Wnt ligands and β-catenin in the crown epithelium postnatally.^29^ When epithelial β-catenin was deleted postnatally, the experimental mice exhibited ectopic incisors, which were short and blunt with malformed enamel.^29^ Similarly, animal studies on large animals, such as miniature pigs, support that the dynamic expressions of Wnt ligands and inhibitors may form the potential Wnt signaling gradient, thus contributing to cusp patterning and crown calcification.^30^ These results confirmed a significant role for Wnt/β-catenin signaling during amelogenesis.

In mouse M1, rapid tooth growth and root elongation begin on PN6.^23^ During the transition/root stage of root formation in mice, we detected highly active Wnt/β-catenin signaling in odontoblast-lineage cells, HERS cells, and periodontal ligament cells.^31^ We observed activated Wnt/β-catenin signals in the apical papilla and dental sac from PN7. Subsequently, we noted a decline of signaling activity in these cells when they received terminal differentiation from PN15 onward and eventually disappeared the signaling in M1. Axin2, known as a component of the β-catenin degradation complex, was also tightly linked with the developing roots. We mainly localized Axin2 expression surrounding the root sheath and dental papilla on PN10.^11^ However, unlike the high Axin2 levels in the dental papilla/pulp (as this part proceeded to develop and differentiate), the Axin2 expression was downregulated in the crown odontoblast lines.^11^ The canonical Wnt signaling may also be indispensable to the differentiation and maturation of odontoblasts, whereas the inactivation of canonical Wnt activity probably act a pivotal role in terminal differentiation. Specifically, the tissue-specific ablation of β-catenin in developing odontoblasts led to rootless molars, whereas the constitutive stabilization of β-catenin in the dental mesenchyme produced molars with short roots and thick cementum.^13,14^ Further insight of the regulatory mechanism of Wnt/β-catenin in cellular differentiation and matrix secretion will be needed during many phases of postnatal root development in future studies.

Within the constraints and limitations of our study, a few tissues in the M1, where BMP and/or Wnt activity was expected, were not stained. This might be because (1) the expression of BMP and/or Wnt signaling are so transient that it is hard to upregulate β-galactosidase expression, (2) the window of signaling activation is missed, or (3) BMP and/or Wnt signaling in these tissues worked in a noncanonical signaling cascade way.

## Conclusions

In short, the results of this study showed that BMP and Wnt signaling activities exhibited different and dynamic patterns during mouse M1 root development. The position of activation of the BMP/Smad signal pathway was located more coronally in the early stage, which then gradually expanded as root elongation proceeded and was associated with blood vessels in the pulp and DAC tissues in the later stage. However, Wnt/β-catenin signaling was highly concentrated in the mesenchyme below the cusps in the early stage, gradually expanded to regions around the root at the transition/root stage, and then disappeared nearly the later stage. These findings emphasized the importance of spatial and temporal epithelial-mesenchymal signaling, such as BMP and Wnt signaling pathways, for postnatal dentinogenesis, as well as provided some clues for future studies to precisely explore the cellular and molecular mechanisms that regulate tooth root development.
